# Polyamines of unique structure are integrated in *Synura echinulata *biosilica

**DOI:** 10.1007/s00216-025-05891-3

**Published:** 2025-05-07

**Authors:** Oliver Reinke, Susanne Machill, Eike Brunner

**Affiliations:** Chair for Bioanalytical Chemistry, TU Dresden, Dresden, 01062 Germany

**Keywords:** Biomineralization, Biosilica, LCPAs, Mass spectrometry, *Synurales*, Diatoms

## Abstract

Unicellar, biomineralizing algae like diatoms or *Synurales* are ubiquitous in various habitats all over the world and have an outstanding role in different biogeochemical cycles. They are well known for their elaborate nanopatterned cell structures consisting of amorphous biosilica, which is intracellularly synthesized. Special biomolecules assist in the silica formation. In particular, species-specific *long-chain polyamines* (LCPAs) are commonly found in diatom biosilica and seem to play a special role due to their ability to self-assemble and induce silica precipitation. In contrast to diatoms, no species from the order *Synurales* have been tested yet for the presence of LCPAs. Therefore, the present work deals with the analysis of *Synura echinulata* biosilica using a novel HPLC-HR-MS/MS method. The presence of unique LCPAs is shown, and their structure is elucidated via MS/MS experiments. LCPAs from *S. echinulata* are based on amino butyl repeat units—in contrast to previously described LCPAs from other organisms, which are mostly based on amino propyl repeat units. The ubiquitous presence of LCPAs in biomineralizing species strongly indicates a general role of LCPAs in silica biomineralization.

**Graphical Abstract:**

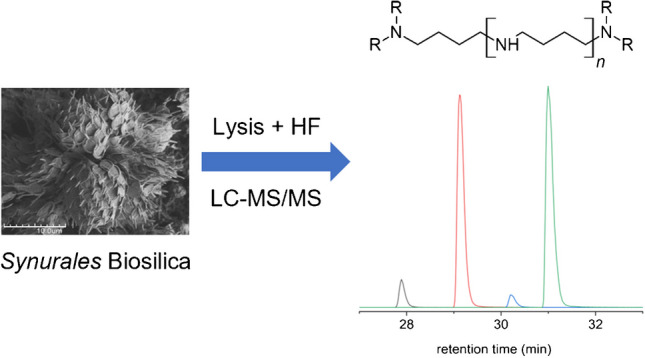

**Supplementary Information:**

The online version contains supplementary material available at 10.1007/s00216-025-05891-3.

## Introduction

Silica biomineralization is the genetically controlled formation of silica-based mineral structures by living organisms [1]. It occurs, for example, in various algae but also in higher plants like equisetum, stinging nettle, and others. A prominent example of biomineralizing algae are the ubiquitously occurring unicellular diatoms, a major part of the phytoplankton [2–6]. Their siliceous cell walls, the so-called frustules (diatom biosilica) exhibit an impressive structure with species-specific patterns at the micrometer and even nanometer scales [7] with potential applications, e.g., in catalysis, optical technology, or as separation membranes for biomolecules [8–15]. Over the past decades, diatoms have often served as model organisms for silica biomineralization studies. Various biosilica-associated biomolecules have meanwhile been identified [2, 16–24]. A particularly interesting class of tightly silica-associated biomolecules is the longchain polyamines (LCPAs) discovered by Kröger and Sumper [25, 26]. In vitro experiments have shown that LCPAs, with their positively charged amine groups, can self-assemble in the presence of suitable counterions and trigger silica precipitation [25, 27–39]. Tightly biosilica-attached LCPAs have meanwhile been found in various diatom species [25]. Solidstate NMR spectroscopic studies and their presence in diatom biosilica-containing sediments suggest that LCPAs are even embedded into the silica phase [37, 40–43]. Their molecular structure is species-dependent, especially with respect to chain length and degree of methylation, i.e., the studied species exhibit characteristic individual sets of LCPAs [25]. Furthermore, the presence of LCPAs in other biomineralizing organisms like sponges [44] and thermophiles [45, 46] has meanwhile been demonstrated, although structural differences to LCPAs from diatoms were observed [25, 26, 30, 44–46]. Recently, the presence of LCPAs even in silicified *Bacillus cereus* spores was shown for the first time [47]. It is tempting to speculate that LCPAs are a general recurring motif in silica biomineralization because such different organisms incorporate them into biosilica.

**Fig. 1:**
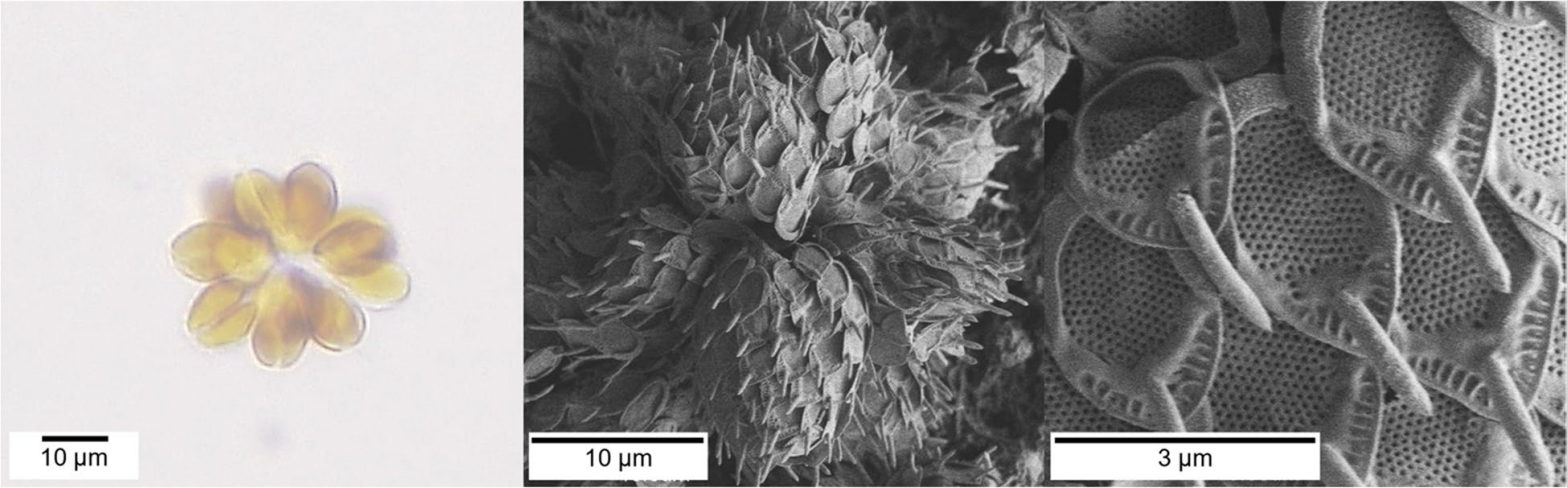
Microscopic images of *S. echinulata*. Left: light-microscopic image of a *S. echinulata* cell colony. Middle: SEM image of *S.* *echinulata* cells. Note the characteristic silica scales covering the cell surface. Right: SEM image of *S. echinulata* silica scales with characteristic spines and pore patterns

Apart from diatoms, there are other biosilica-forming algae, such as *synurids *(order *Synurales*). They occur in various habitats — predominantly in freshwater. Both diatoms and *Synurales* belong to the pigmented *Heterokont* algae, but their lineage diverged around 400 Mya [48, 49]. Diatoms probably originated about 250 Mya during the Permian–Triassic mass extinction event. They quickly diverged and radiated, as indicated by fossil records and molecular clock studies — similar to the *Chrysophyceae* [48]. In the following, the genus *Synura* (order *Synurales*, family *Synuraceae*, class *Chrysophyceae*) appeared approximately 145 Mya. *Synura* later divided into the three sections *Synura*, *Curtispinae*, and *Petersenianae* about 110 Mya [50, 51]. The species *Synura echinulata*, which is studied in the present work, is classified into the genus *Synura*, more precisely into the section *Curtispinae.* It exhibits species-specific silica scales, aggregates into spherical colonies (cf. Fig. 1), and is even able to grow without silicic acid, thus forming cells without siliceous scales [50]. This is in contrast to diatoms, which require silicic acid for cell division and growth. Furthermore, diatom biosilica is structurally different from the siliceous *synurid* scales [52–55]. In analogy to the diatoms, s*ynurales* take up silicic acid from surrounding water via silicic acid transport (SIT) proteins, and silica formation takes place in an SDV (silica deposition vesicle). *Synurales* scales are shaped by an intracellular folding process — involving at least two membrane systems and two actin microfilaments [56–58]. However, the molecular machinery involved in biosilica formation by *synurales* is almost unknown* —* in contrast to diatoms (see above)*.* The presence of LCPAs in *synurales* has not yet been tested. Given their importance in other biosilica-forming organisms, the present work aims to answer the question of whether or not LCPAs are also present in the siliceous scales of the *synurales* species *Synura echinulata*.

## Materials and methods

### Cultivation

The algae *Thalassiosira pseudonana* (strain CCMP 1335) and *Synura echinulata* (strain SAG 15.92) were chosen for the experiments. *Thalassiosira pseudonana* was cultured in artificial seawater (ASW) [59]. Each 20 L culture was inoculated with 100 mL of a dense cell culture. The growth medium was slowly stirred without active aeration. pH was kept between 7.5 and 8.5. After 9–12 days, the cultures were ready for harvest with cell densities around 1.3 $\times$ 10^6^ mL^−1^.

*Synura echinulata* was obtained from “Sammlung von Algenkulturen der Universität Göttingen” (Culture Collection of Algae at Göttingen University, Germany, SAG) and cultivated in WC medium according to Guillard and Lorenzen [60]. Inoculation was done by adding 150 mL of dense cell culture into 20 L of WC medium. The growth medium was slowly moved and actively aerated with compressed air that was previously filtered with a 0.22 µm sterile filter. The pH was kept between 6.5 and 7.5. After 35–45 days, the cultures were harvested.

Both algae were cultivated in sealed polycarbonate containers (Nalgene) filled with 20 L sterile medium. Cultures were grown in an incubator (RuMed 1301, Rubarth Apparate GmbH, Laatzen, Germany) with a constant temperature of 20 °C and a 12-h/12-h day/night cycle. During cultivation, silicic acid concentration in the medium and cell density were monitored via molybdenum blue assay (MBA) [61, 62] and an automated Cell Counter (CellDrop FL, DeNovix, Wilmington, DE, USA). Cells were harvested in the late exponential growth phase by centrifugation with 4000 $\times$ g for 25 min (Heraeus Megafuge 40, swinging bucket rotor TX 750, Thermo Scientific, Waltham, MA, USA).

### Isolation of biosilica and extraction of LCPAs

In the present work, the isolation process was performed similarly to Bridoux et al. [40, 43, 63]. First, the cell pellet of a 20 L culture was lysed following the procedure established by Kröger et al. [25]. The cell pellet was mixed with 20 mL of lysis buffer (2.0% (w/v) sodium dodecyl sulfate (SDS) and 0.1 M ethylenediaminetetraacetic acid (EDTA) at pH 8.0) [25]. The mixture was heated at 95 °C for 10 min. After cooling, the lysate was centrifuged (2000 $\times$ g, 15 min, Universal 320R, swinging bucket rotor 1494, Andreas Hettich GmbH & Co. KG, Tuttlingen, Germany). The resulting biosilica was washed by mixing it with ultrapure water and centrifuged, as described above. After 5–6 washing cycles, the supernatant was colorless. The obtained material was then lyophilized.

To release the embedded LCPAs, the lyophilized cell wall material was dissolved by the addition of 2 mL concentrated HF (40%) in a Teflon reaction vessel. The resulting solution was evaporated in a constant nitrogen stream at 100 °C over 2 days. The remaining material was redissolved in 750 µL ultrapure water. This solution was centrifuged for 15 min at 15,000 RPM, and the supernatant was centrifuged again (Universal 320R, fixed angle rotor 1420-B, Andreas Hettich GmbH & Co. KG, Tuttlingen, Germany). Before injection into the HPLC–MS, the solution was filtered through a 0.45 µm centrifugal filter (Modified Nylon, Low protein binding, VWR International, Radnor, PA, USA) to remove undissolved particles.

### Reductive methylation of extracted LCPAs

To differentiate between different methylation degrees and isomeric structures of the LCPAs, a reductive methylation was conducted. For this, the procedure of Jentoft et al. [64] was adapted by adding 100 µL of NaCNBH_3_ solution (0.1 M) to the re-dissolved LCPAs, followed by intense mixing. Afterwards, 100 µL HCOH (0.1 M) was added, and the mixture was stored in the fridge overnight. The resulting permethylated LCPAs were then directly injected in the LC–MS system for analysis.

### HPLC–MS analysis for characterization of isolated LCPAs

For subsequent analysis of the LCPA-containing solution, an Agilent HPLC 1260 Infinity chromatography system coupled to an Agilent 6538 UHD Accurate-Mass Q-TOF mass spectrometer was used. Ionization was conducted with an ESI source and a Polaris 3 C18-ether separation column (150 mm $\times$ 3.0 mm, 3 µm) was used. The HPLC–MS method was based on procedures established by Bridoux et al. which were adapted and further developed in our experiments [40, 43, 63]. Separation was achieved by using a flow rate of 0.4 mL/min and a gradient of 0.1% (v/v) HFBA in water (solvent A) and 0.1% HFBA in ACN (solvent B). Solvent B was ramped from 0 to 80% in 60 min followed by a 15-min post-time with the initial solvent (100% A). Besides, the column temperature was held at 40 °C, and mass spectrometric parameters were 1.0 spectrum/s, dry gas (nitrogen) 12 L/min at 350 °C, nebulizer gas 60 psig, capillary voltage 3500 V, fragmentor voltage 100 V, skimmer voltage 45 V, and octapol voltage 400 V. m/z values were scanned from 50 to 2000. Fragmentation experiments were conducted with the same parameters for HPLC–MS with a collision energy between 10 and 50 eV, depending on the chain length of the fragmented LCPA.

## Results and discussion

Different methods exist for the extraction and analysis of biosilica-attached LCPAs. Sumper et al. isolated LCPAs via cation exchange chromatography and size exclusion chromatography [26]. Analysis was then performed by MALDI-TOF mass spectrometry. This technique leads to excellent results but requires an elaborate isolation and purification procedure. Furthermore, no high-resolution mass spectra are detected [26]. Bridoux et al. directly injected the LCPA-containing solution into an HPLC–MS/MS system equipped with a C8 column and an ESI-source. By using HFBA as an ion pair reagent in the HPLC, the separation of different LCPA fractions, as well as the elucidation of the LCPAs structure, became possible [40, 43, 63].

For the present work, a novel HPLC-HR-MS/MS method was established and verified as described in detail in ESI. It allows on-line LCPA separation and subsequent characterization, in contrast to the MALDI-TOF–MS analyses of Sumper et al., which lack the on-line separation step [26]. Different from the method established by Bridoux et al., a more common C18 column is used for our experiments in combination with a high-resolution (HR) mass spectrometer. The latter allows identification of molecular fragments based on highly accurate ion masses [40, 43, 63]. That means the developed HPLC-HR-MS/MS method exploits the HF-stability of LCPAs to minimize the number of sample preparation steps and hence the analyte loss. It additionally offers a straightforward on-line separation, as well as a faster and more accurate analyte identification and structure elucidation by using a HR-MS. To gain even more structural insight, a reductive methylation was conducted, as in the work of Sumper et al. and Bridoux et al. [26, 63]. To validate the novel HPLC-HR-MS/MS method, the well-known previous results obtained by Sumper et al. for *T. pseudonana* were reproduced. All obtained chromatograms, MS, and MS/MS spectra perfectly matched with previously published data (for further information, see Fig. S1-5, Table S1). After its successful implementation and validation, the developed novel method served to investigate the biosilica scales of *S. echinulata*.

### Morphological analysis of biosilica from *Synura echinulata*

The cells of *Synura echinulata* aggregate into spherical colonies (Fig. 1). Each *S. echinulata* cell is covered in species-specific silica scales that differ slightly depending on their position on the cell surface. The scales exhibit a characteristic spine as well as a perforated area at the distal end and a thickened rim at the proximal end. Besides, an ordered, labyrinthine pore pattern can be observed on the cell surface (Fig. 1) [50, 56, 58].

### Analysis of *Synura echinulata* biosilica

**Fig. 2:**
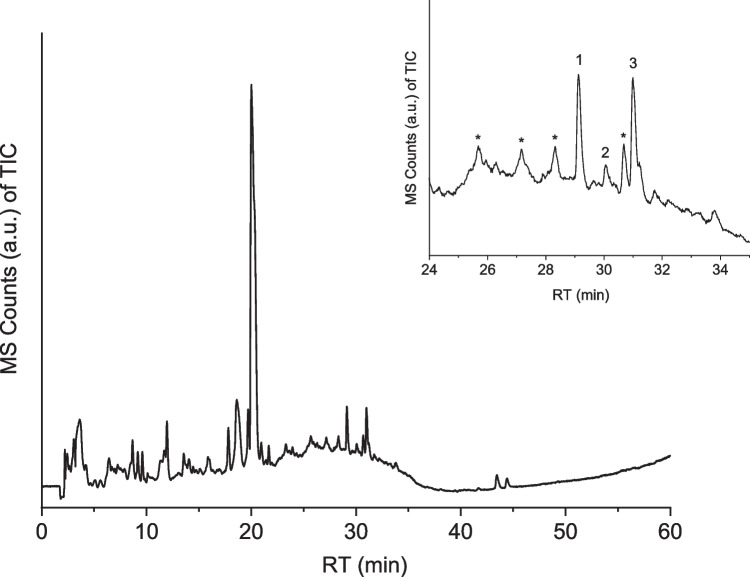
HPLC–MS (TIC) chromatogram of the re-dissolved remnant after HF treatment of lysed *S.* *echinulata* cell walls. Inset: retention time window characteristic for LCPAs with indicated fractions 1–3. *Analysis of these peaks revealed that they are not due to LCPAs

The harvested *S. echinulata* cell pellet was lysed, and the resulting biosilica was dissolved in HF, as described in the experimental section. HPLC–MS analysis reveals the chromatogram shown in Fig. 2. It exhibits several chromatographic peaks in the retention time window where LCPAs are expected, similar to those in *T. pseudonana.* It was found that the two most intense peaks (fractions 1 and 3) are indeed due to LCPA signals, as indicated by their exact mass determination (Fig. 2).

**Fig. 3:**
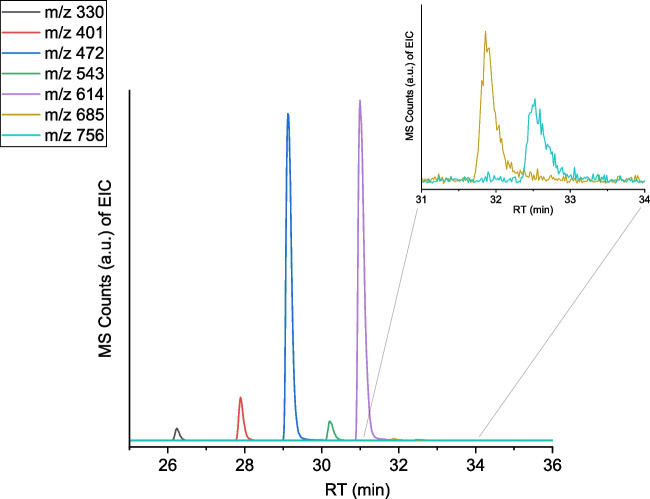
Extracted ion chromatogram (EIC) of the found homologous LCPA series. Inset: enlarged retention time window of longer LCPAs

One of the smaller peaks (peak 2) is also identified as a minor LCPA signal, whereas the other peaks cannot be assigned to LCPAs. Extraction of the m/z values 472.5049, 543.5817, and 614.6525 revealed three peaks (Fig. 3). The mass difference of about 71.07 mass units between these signals strongly points towards a homologous series of LCPAs with a [C_4_H_9_N] repeat unit. Consequently, calculated m/z values of further elements of the homologue series were isolated via extraction of their ion chromatogram, resulting in the discovery of additional LCPAs with m/z 330.3597, 401.4175, 685.7248, and 756.7988 (Fig. 3). By comparing the integral intensity of the extracted ion signals, conclusions about their relative abundance are possible. Figure 3 shows that LCPAs with m/z = 472.5049 and 614.6525 are dominating. They correspond to LCPAs with *n* = 5 and *n* = 7 repeat units. LCPAs with m/z = 330.3597, 401.4175, 543.5817, 685.7248, and 756.7988 are less abundant, i.e., their relative amount is at least ten times lower.

**Fig. 4:**
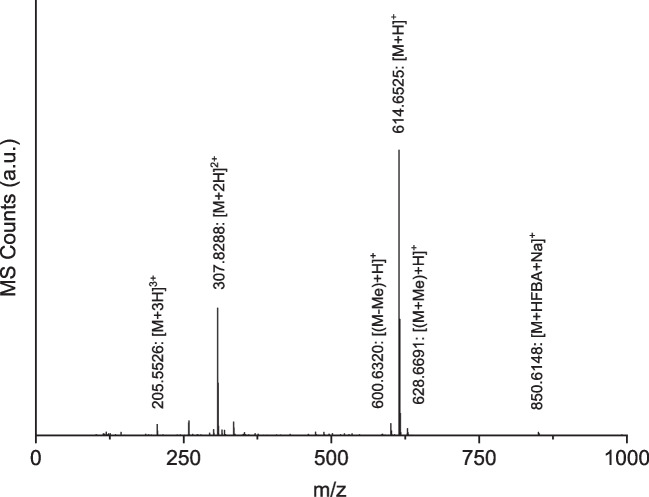
MS spectrum of LCPA fraction 3 (RT 31.00 min) showing differently charged LCPA ions, adducts, and variants of different methylation degree

In addition to the singly charged LCPA ions, two- and three-fold charged ions can be found as well as adducts of LCPA and the ion-pair reagent HFBA (Fig. 4). Moreover, ion signals with a difference in m/z of about 14.016 mass units relative to the main LCPA signals with m/z = 472.5049, 543.5817, and 614.6525 were detected. These intervals indicate the existence of versions of the main LCPAs with varying methylation patterns either at the terminal amine functions or at internal amine groups. The m/z difference of 14.016 results due to a net-addition of a methylene (CH_2_) group by an exchange of a hydrogen residue with a methyl group at an amine function. Regarding their frequency of occurrence, their signal intensity is significantly lower than the intensity of the corresponding LCPA.

All detected m/z values due to LCPAs are summarized in Table 1. Despite the observed main compounds, homologues with a higher chain length or different methylation patterns could exist but cannot be detected due to their low signal intensity. Based on the results discussed so far, a preliminary structure with a polyamine base, a [C_4_H_9_N] repeat unit, and singly methylated terminal amine groups can be proposed for the found LCPA series.

**Table 1:** Summary of all found LCPAs in the cell walls of *S. echinulata* with *n*, number of repeat units, and *i*, number of methyl groups relative to the most abundant LCPA with equal chain length (*i*_main_ = 0)

Fraction	RT (min)	***n*** (***i***)	m/z (measured)	Composition	m/z (theor.)	Δ m/z
-	26.23	3 (0)	330.3582	[C_18_H_43_N_5_ + H]^+^	330.3592	0.0010
-	27.89	4 (0)	401.4318	[C_22_H_52_N_6_ + H]^+^	401.4327	0.0009
-	27.97	4 (1)	415.4471	[C_23_H_54_N_6_ + H]^+^	415.4483	0.0012
1	29.00	5 (-1)	458.4893	[C_25_H_59_N_7_ + H]^+^	458.4905	0.0012
	29.12	5 (0)	472.5049	[C_26_H_61_N_7_ + H]^+^	472.5062	0.0013
	29.24	5 (1)	486.5206	[C_27_H_63_N_7_ + H]^+^	486.5218	0.0012
	29.35	5 (2)	500.5357	[C_28_H_65_N_7_ + H]^+^	500.5375	0.0018
2	30.20	6 (0)	543.5817	[C_30_H_70_N_8_ + H]^+^	543.5797	0.0020
	30.33	6 (1)	557.5939	[C_31_H_72_N_8_ + H]^+^	557.5958	0.0019
3	30.91	7 (-1)	600.6362	[C_33_H_77_N_9_ + H]^+^	600.6375	0.0013
	31.00	7 (0)	614.6525	[C_34_H_79_N_9_ + H]^+^	614.6532	0.0007
	31.13	7 (1)	628.6672	[C_35_H_81_N_9_ + H]^+^	628.6688	0.0016
-	31.86	8 (0)	685.7248	[C_38_H_88_N_10_ + H]^+^	685.7267	0.0019
-	32.53	9 (0)	756.7988	[C_42_H_97_N_11_ + H]^+^	756.8002	0.0014

**Fig. 5:**
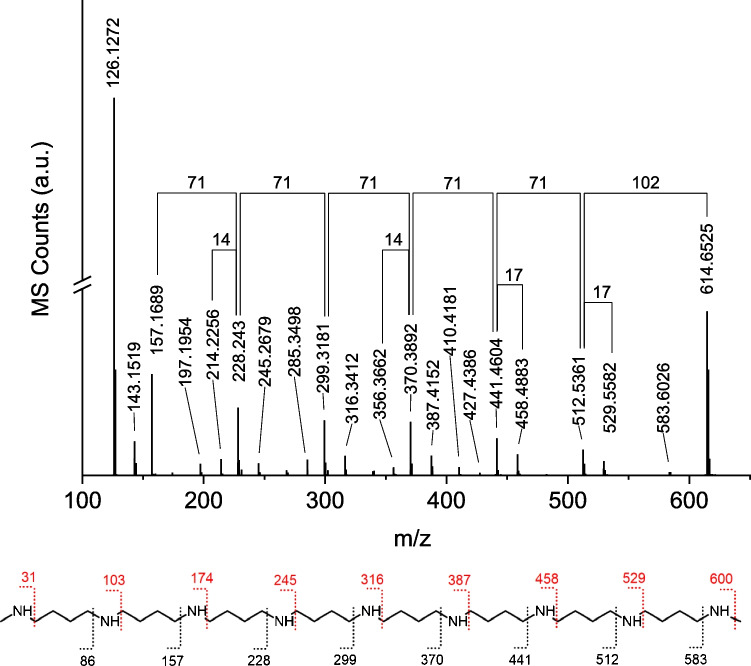
MS/MS fragmentation spectrum (top) and fragmentation pattern (bottom) of m/z 614.6525 (LCPA fraction 3). It clearly reveals the characteristics of LCPA fragmentation, like the loss of multiple repeat units (∆ m/z 71.07) as well as the loss of methylene (∆ m/z 14.016) or ammonia (∆ m/z 17.027)

To verify this proposed structure, the ions with m/z values 472.5049, 543.5817, and 614.6525 were further analyzed in MS/MS-fragmentation experiments to gain conclusive structural evidence. To this end, a fragmentation voltage is applied after the isolation of a certain m/z value, leading to bond breakage and thus the formation of characteristic fragment ions. The fragmentation spectrum of m/z = 614.6525 (Fig. 5) clearly shows the characteristics of LCPAs. First, a common m/z interval is 71.07. This is indicative of the loss of a [C_4_H_9_N] fragment, which could be either a methylamino propyl unit or an amino butyl unit due to their isomeric nature. It is found multiple times in the spectrum (Fig. 5), supporting the assumption of [C_4_H_9_N] as the repeat unit. The most dominant fragment ion with m/z 126.1272 could be assigned to a [C_8_H_16_N]^+^ fragment ion, which may result from the loss of ammonia from an amino butyl-based dimer. Furthermore, the m/z difference of 102.1164 between the molecular ion and one of the first fragment ions at m/z = 512.5361 can be assigned to the loss of a [C_5_H_14_N_2_] fragment, which could be either a N1,N2-dimethyl-1,3-diamino propyl unit (also found in fragmentation spectra of LCPAs from *T. pseudonana* (see ESI)) or a N1-methyl-diamino butyl unit*.* Further fragment ion m/z differences of 14.016 and 17.027 indicate methylene groups or ammonia. These are also common mass losses for LCPAs.

In addition, the ions with m/z = 472.5049 (LCPA fraction 1) and 543.5817 (LCPA fraction 2) were fragmented to clarify their structure. Both spectra (Fig. S6 and S7) feature common characteristics of LCPA fragmentation, as described above. In comparison to the fragmentation spectra of m/z = 614.6525, the spectra of m/z = 472.5049 and 543.5817 show a different number of losses, with m/z 71.07. Taken together, m/z = 472.5049 and 543.5817 can thus be identified as LCPAs with a homologue structure to m/z 614.6525, with fewer repeat units.

To unambiguously differentiate between the isomeric structures of LCPAs, a further experiment called reductive methylation is necessary. In this reaction, all primary and secondary amine functions of the naturally occurring LCPAs are transferred into tertiary amine groups by N-methylation. Consequently, the MS spectra, as well as the MS/MS spectra of the N-methylated LCPAs, should show strongly reduced complexity. Also, by comparing the expected and measured results of the MS experiments, the hypothesized LCPA structures can be tested. As expected, the chromatogram peaks in LC–MS analysis of the N-methylated LCPAs are shifted to higher retention times, compared to the unmodified ones, due to their higher methylation degree and thus lower polarity (Fig. S8). Regarding their MS spectra, a difference between the m/z of naturally occurring LCPAs and the m/z of N-methylated LCPAs is observed (Fig. S9, S10, S11). This m/z difference allows one to calculate the number of added methyl groups per molecule, and therefore, the number of former primary and secondary amine functions can be concluded (Table 2).

**Table 2:** Calculation of the number of added methyl groups per LCPA molecule by reductive methylation

m/z _native_	m/z_methylated_	Δ m/z	Δ m/z : m (CH_2_) (CH_2_: m/z 14.0157)	Number of added methyl groups
472.5039	570.6147	98.1108	7.0001	7
543.5817	655.6996	112.1179	7.9995	8
614.6525	740.7874	126.1349	8.9996	9

**Fig. 6:**
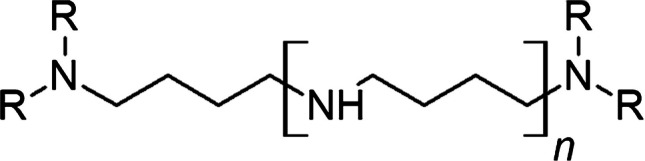
General structure (*R* = -CH_3_, -H; *n* is the number of repeat units, ranging from 2 to 9) of the discovered homologous LCPA series in *Synura echinulata*. The most abundant LCPAs exhibit one terminal N-methyl group at each end

Based on the above-described results about the molecular structure, a seven- to ninefold N-methylation per molecule is only possible if the amine groups within the chain are secondary amines. This fact and the determined molecular sum formula [C_4_H_9_N] leads to the unambiguous conclusion of amino butyl groups as the repeat unit. Combining the information derived by MS and MS/MS measurements as well as the reductive methylation experiments, the molecular structure below is obtained as the general structure of the homologous LCPA series in *Synura echinulata* (Fig. 6).

The proposed structures are further substantiated by the observation that the measured exact masses agree very well with the expected exact masses for the derived structures within the limits of the measurement error (Table 1) and by the perfect match of the numbers of amine functions and added N-methylations in the reductive methylation experiments.

Finally, the proposed structures were again confirmed by LC–MS/MS fragmentation analyses of the reductively methylated LCPAs. The obtained fragmentation spectra of LCPA fractions 1–3 are shown in Fig. S9-S11 (see ESI). As a characteristic example, in the fragmentation spectrum of m/z 740.7874 (corresponding N-methylated LCPA to m/z 614.6525, LCPA fraction 3, Fig. S11), we observe a common m/z interval of 85.089 originating from the loss of an N-methyl amino butyl unit. In addition to this dominating fragment, an m/z interval of 130.145 is noticeable between the quasi-molecular ion and the first fragment ion, which can be assigned to a [C_7_H_18_N_2_] fragment. Furthermore, a fragment with m/z 100.112 indicates a [C_6_H_14_N]^+^ fragment. All observed mass losses are expected and thus fully support the conclusion that m/z 740.7874 is the fully N-methylated form of m/z 614.6525 (Fig. S11) with the above described LCPA structure (Fig. 6).

This fragmentation pattern can also be observed in the spectra of m/z 570.6147 and 655.6996 but at varying numbers of repeat unit losses caused by the different chain lengths. This characteristic fragmentation pattern further corroborates the proposed general structure of the LCPA series.

In summary, a homologous series of LCPAs could be identified in the biosilified scales of *Synura echinulata*. Furthermore, the LCPA structure was determined via different MS and MS/MS measurements as well as through reductive methylation experiments.

### Comparison of LCPAs from different organisms

LCPAs from other organisms are species-specific and variable, especially with respect to their chain length and methylation degree. Here, for the first time, we show the presence of LCPAs in biosilica from a *Synurales* species. Furthermore, the analyzed species *Synura echinulata* exhibits putrescine-based LCPAs with an amino butyl repeat unit. So far, this unusual motif is only described once in a diatom and sediments from Lake Baikal [67]. But the LCPAs found in *S. echinulata* are protonated in contrast to the methylated structures found by Annenkov et al. The chain length of the LCPAs of *Synura echinulata* is typically found between 2 and 9 repeat units, i.e., they are shorter than typical diatom LCPAs with up to 20 repeat units [25–27, 29, 30]. Furthermore, LCPAs from *Synura echinulata* lack internal methylation, similar to LCPAs of diatoms like *T. pseudonana* [26]. The special repeat unit could have an impact on the biomineralization process because longer internal alkyl chains can lead to less polar LCPA molecules. Less polar LCPAs will, however, exhibit a more pronounced self-assembly tendency and thereby a stronger microphase separation, which is crucial for the precipitation of biosilica [65].

The comparison of LCPAs from algae with LCPAs originating from bacteria, archaea, or sponges shows some clear distinctions [44]. (Hyper-) Thermophilic bacteria and archaea possess shorter polyamines (penta- and hexa-amines) than diatoms, and some polyamine structures present in these species are branched [45, 46, 66]. Furthermore, the LCPAs found in bacteria, archaea, and sponges appear to be completely non-methylated, which is a major difference to the methylation patterns found in diatoms, but similar to the LCPAs found in *Synura echinulata*. Additionally, LCPAs found in *B. cereus* seem to be considerably longer than LCPAs found in algae [47].

## Conclusions

In this study, the existence of LCPAs embedded into the biosilica scales of the *Synurales* species *Synura echinulata* was described for the first time. A novel HPLC–MS/MS method was developed for the structural analysis of the silica-attached LCPAs. It uses a standard C18 column and can separate LCPAs and determine masses with high accuracy since a high-resolution mass spectrometer is used. This method was validated by fully reproducing the previously known LCPA structure of *T. pseudonana* diatom biosilica. Analysis of cell walls from *S.* *echinulata* revealed unique, new biosilica-attached LCPAs based on amino butyl repeat units. This is different from common LCPAs in other biosilica-producing organisms, such as diatoms, sponges, bacteria, or archaea. So far, amino butyl-based LCPAs have only been described once in a diatom and sediments from Lake Baikal [67]. It is tempting to speculate that the amino butyl repeat unit may be more widespread in biosilica-forming organisms than assumed before. Finally, the observation of LCPAs in *Synura echinulata* and various other biosilica-forming organisms increasingly indicates that LCPAs may be a general feature of silica biomineralization processes. For further studies, it would be interesting to shed light on whether the novel LCPAs could possibly be bound to proteins like the silaffins in diatoms or if they preferentially exist as single molecules.

## Supplementary Information

Below is the link to the electronic supplementary material.

ESM 1 Supplementary Material 1: Results of the method verification by analysis of the LCPAs from *T. pseudonana*, Additional chromatographic, MS and MS/MS data of the analysis of LCPAs from *Synura echinulata *(PDF 1.18 MB)

## Data Availability

All data are available from the authors by request.
