# Correction to: Polyamines of unique structure are integrated in *Synura echinulata* biosilica

**DOI:** 10.1007/s00216-025-05978-x

**Published:** 2025-06-24

**Authors:** Oliver Reinke, Susanne Machill, Eike Brunner

**Affiliations:** Chair for Bioanalytical Chemistry, TU Dresden, Dresden, 01062 Germany

**Correction to: Analytical and Bioanalytical Chemistry**

10.1007/s00216-025-05891-3

The original version of this article unfortunately contained mistakes.

Corrections are:

Abstract

*Previous version:* “LCPAs from *S. echinulata* are based on amino butyl repeat units — in contrast to all previously described LCPAs from other organisms, which are based on amino propyl repeat units.”

*Replacement:* “LCPAs from *S. echinulata* are based on amino butyl repeat units — in contrast to previously described LCPAs from other organisms, which are mostly based on amino propyl repeat units.”

Results and Discussion, Comparison of LCPAs from different organisms

*Previous version:* “This was not discovered in biosilica from other organisms.”

*Replacement:* “So far, this unusual motif is only described once in a diatom and sediments from Lake Baikal [67]. But the LCPAs found in *S. echinulata* are protonated in contrast to the methylated structures found by Annenkov et al.”

Conclusions

*Previous version:* “This is different from LCPAs in other biosilica-producing organisms, such as diatoms, sponges, bacteria, or archaea. The results of this study expand the knowledge about naturally occurring LCPAs regarding their structural variability.”

*Replacement:* “This is different from common LCPAs in other biosilica-producing organisms, such as diatoms, sponges, bacteria, or archaea. So far, amino butyl-based LCPAs have only been described once in a diatom and sediments from Lake Baikal [67]. It is tempting to speculate that the amino butyl repeat unit may be more widespread in biosilica-forming organisms than assumed before.”

Added reference:

67. Annenkov VV, Verkhozina ON, Zelinskiy SN, Shishlyannikova TA, Bridoux MC, Danilovtseva EN. Unusual Polyamines from Baikalian Diatoms. ChemistrySelect. 2018;3:9708–9713. 10.1002/slct.201802032

The original article has been corrected.

