# Supplementary material for: Polyamines of unique structure are integrated in Synura echinulata biosilica
Source: Anal Bioanal Chem. 2025 May 7;417(16):3675–84. doi: 10.1007/s00216-025-05891-3 (PMC12206195; doi:10.1007/s00216-025-05891-3)
Supplement: Supplementary file 1 — Supplementary Material 1: Results of the method verification by analysis of the LCPAs from T. pseudonana, Additional chromatographic, MS and MS/MS data of the analysis of LCPAs from Synura echinulata (PDF 1.18 MB) [file 216_2025_5891_MOESM1_ESM.pdf]

Electronic Supporting Information (ESI) for

Polyamines of Unique Structure are Integrated in *Synura echinulata* Biosilica

*Oliver Reinke, Susanne Machill, Eike Brunner**

Chair for Bioanalytical Chemistry, TU Dresden, 01062 Dresden, Germany

ORCID: 0009-0007-9745-1295, 0000-0003-0255-0391, 0000-0003-3511-9899

*Correspondence: eike.brunner@tu-dresden.de

Keywords

Biomineralization, Biosilica, LCPAs, Mass Spectrometry, *Synurales*, Diatoms

Verification of the newly established LC-MS/MS method by analysis of the biosilica from *Thalassiosira pseudonana*

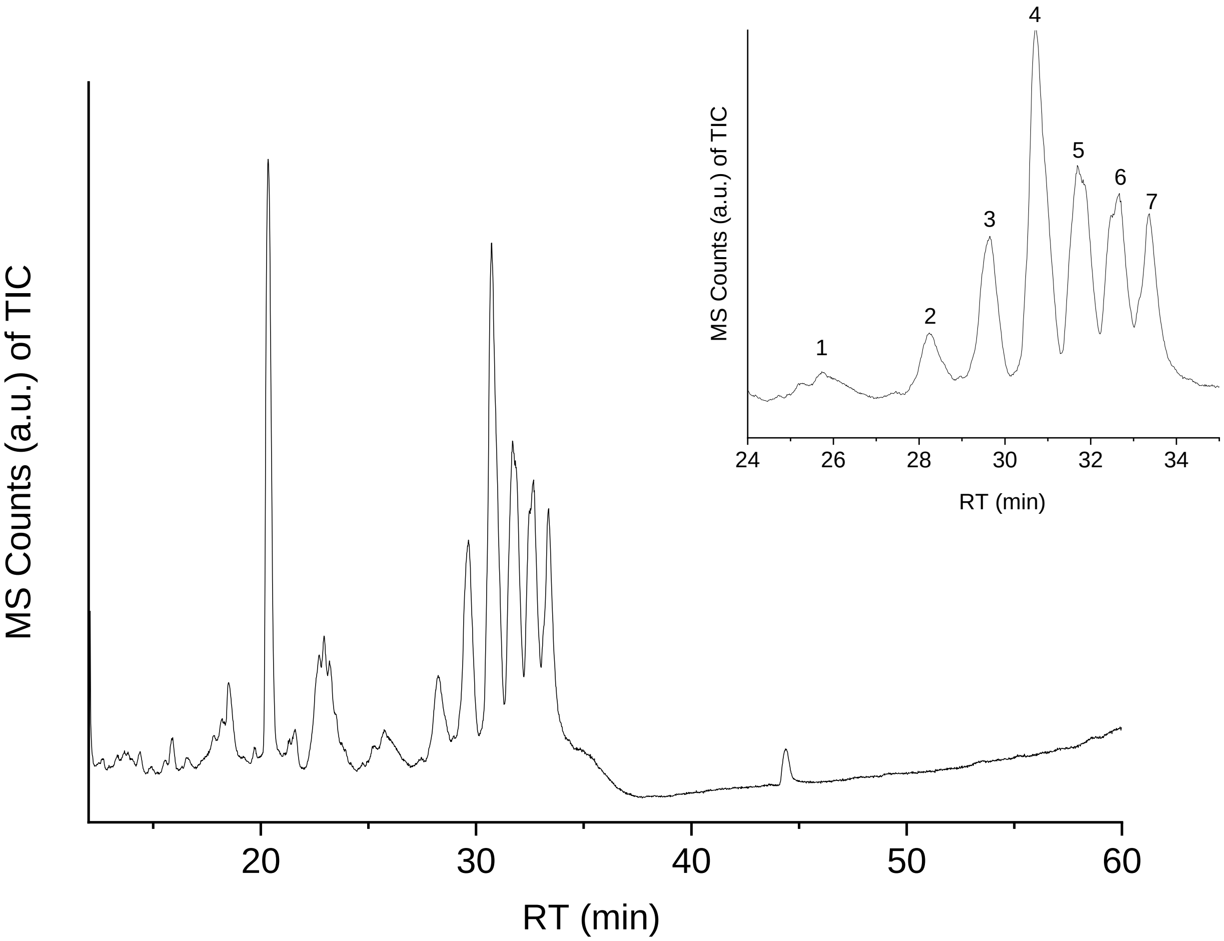

Fig. S1 HPLC-MS (TIC) chromatogram of the redissolved remnant after HF treatment of lysed *T. pseudonana* cell walls. Insert: Interesting LCPA retention time window with LCPA fractions 1-7

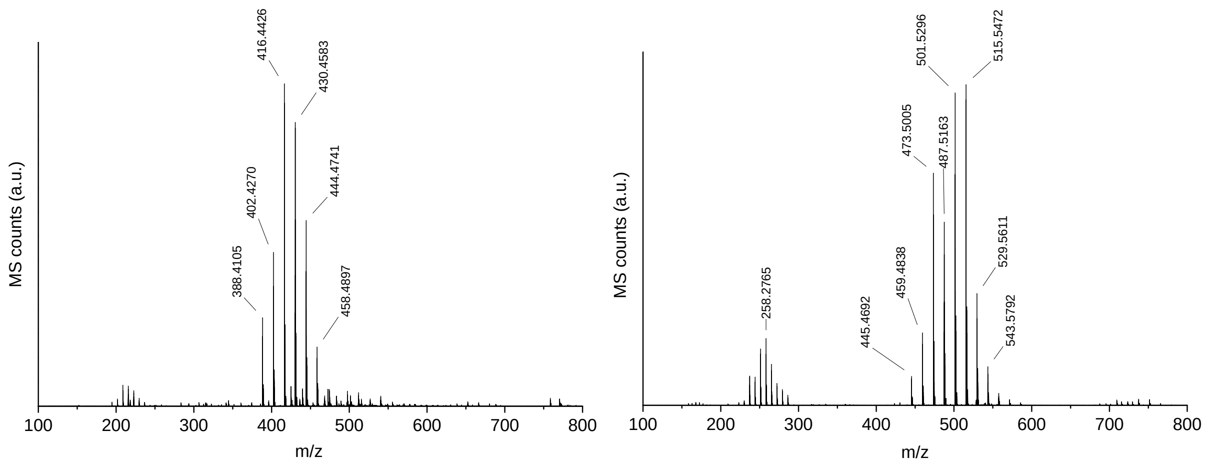

Fig. S2 Mass spectra of LCPA fraction 3 (left) and fraction 4 (right): Both spectra show different LCPA ions (intense signal group), their double charged ions (shifted to smaller m/z values) and LCPA-HFBA adducts (shifted to higher m/z values); Between different fractions m/z values of the ion set increase by about Δ m/z 71.07, which strongly hints at a variation in chain length; Within one fraction the ions of one set differ by a m/z value of about 14.016 which indicates a variation in methylation

Table S1 Summary of LCPAs detected in biosilica of *Thalassiosira Pseudonana*

Fraction	RT (min)	n	m/z (measured)	Composition	m/z (theor.)	Δ m/z
1	25.73	3	246.2648	$C_{12}H_{31}N_5 + H^+$	246.2653	0.0005
			260.2805	$C_{13}H_{33}N_5 + H^+$	260.2809	0.0004
			274.2962	$C_{14}H_{35}N_5 + H^+$	274.2966	0.0004
			288.3116	$C_{15}H_{37}N_5 + H^+$	288.3122	0.0006
			302.3272	$C_{16}H_{39}N_5 + H^+$	302.3279	0.0007
2	28.24	4	331.3534	$C_{17}H_{42}N_6 + H^+$	331.3544	0.0010
			345.3692	$C_{18}H_{44}N_6 + H^+$	345.3701	0.0009
			359.3846	$C_{19}H_{46}N_6 + H^+$	359.3857	0.0011
			373.4004	$C_{20}H_{48}N_6 + H^+$	373.4014	0.0010
			387.4160	$C_{21}H_{50}N_6 + H^+$	387.4170	0.0010
3	29.65	5	388.4114	$C_{20}H_{49}N_7 + H^+$	388.4123	0.0009
			402.4270	$C_{21}H_{51}N_7 + H^+$	402.4279	0.0009
			416.4426	$C_{22}H_{53}N_7 + H^+$	416.4436	0.0010
			430.4583	$C_{23}H_{55}N_7 + H^+$	430.4592	0.0009
			444.4737	$C_{24}H_{57}N_7 + H^+$	444.4749	0.0012

Fraction	RT (min)	n	m/z (measured)	Composition	m/z (theor.)	Δ m/z
3	29.65	5	458.4897	$C_{25}H_{59}N_7 + H^+$	458.4905	0.0008
			472.5049	$C_{26}H_{61}N_7 + H^+$	472.5062	0.0013
4	30.73	6	445.4686	$C_{23}H_{56}N_8 + H^+$	445.4701	0.0015
			459.4844	$C_{24}H_{58}N_8 + H^+$	459.4858	0.0014
			473.5003	$C_{25}H_{60}N_8 + H^+$	473.5014	0.0011
			487.5163	$C_{26}H_{62}N_8 + H^+$	487.5171	0.0008
			501.5317	$C_{27}H_{64}N_8 + H^+$	501.5327	0.0010
			515.5472	$C_{28}H_{66}N_8 + H^+$	515.5484	0.0012
			529.5636	$C_{29}H_{68}N_8 + H^+$	5295640	0.0004
			543.5792	$C_{30}H_{70}N_8 + H^+$	543.5797	0.0005
5	31.69	7	502.5250	$C_{26}H_{63}N_9 + H^+$	502.5280	0.0030
			516.5418	$C_{27}H_{65}N_9 + H^+$	516.5436	0.0018
			530.5577	$C_{28}H_{67}N_9 + H^+$	530.5593	0.0016
			544.5740	$C_{29}H_{69}N_9 + H^+$	544.5749	0.0009
			558.5897	$C_{30}H_{71}N_9 + H^+$	558.5906	0.0009

Fraction	RT (min)	n	m/z (measured)	Composition	m/z (theor.)	Δ m/z
5	31.69	7	572.6051	$C_{31}H_{73}N_9 + H^+$	572.6062	0.0011
			586.6213	$C_{32}H_{75}N_9 + H^+$	586.6219	0.0006
			600.6366	$C_{33}H_{77}N_9 + H^+$	600.6375	0.0009
			614.6523	$C_{34}H_{79}N_9 + H^+$	614.6532	0.0009
6	32.67	8	559.5849	$C_{29}H_{70}N_{10} + H^+$	559.5858	0.0009
			573.6006	$C_{30}H_{72}N_{10} + H^+$	573.6015	0.0009
			587.6163	$C_{31}H_{74}N_{10} + H^+$	587.6171	0.0008
			601.6323	$C_{32}H_{76}N_{10} + H^+$	601.6328	0.0005
			615.6476	$C_{33}H_{78}N_{10} + H^+$	615.6484	0.0008
			629.6632	$C_{34}H_{80}N_{10} + H^+$	629.6641	0.0009
			643.6786	$C_{35}H_{82}N_{10} + H^+$	643.6797	0.0011
			657.6949	$C_{36}H_{84}N_{10} + H^+$	657.6954	0.0005
			671.7097	$C_{37}H_{86}N_{10} + H^+$	671.7110	0.0013
			685.7253	$C_{38}H_{88}N_{10} + H^+$	685.7266	0.0013

Fraction	RT (min)	n	m/z (measured)	Composition	m/z (theor.)	Δ m/z
7	33.37	9	616.6429	$C_{32}H_{77}N_{11} + H^+$	616.6437	0.0008
			630.6589	$C_{33}H_{79}N_{11} + H^+$	630.6593	0.0004
			644.6747	$C_{34}H_{81}N_{11} + H^+$	644.6750	0.0003
			658.6908	$C_{35}H_{83}N_{11} + H^+$	658.6906	0.0002
			672.7061	$C_{36}H_{85}N_{11} + H^+$	672.7063	0.0002
			686.7208	$C_{37}H_{87}N_{11} + H^+$	686.7219	0.0011
			700.7366	$C_{38}H_{89}N_{11} + H^+$	700.7376	0.0010
			714.7521	$C_{39}H_{91}N_{11} + H^+$	714.7532	0.0011
			728.7679	$C_{40}H_{93}N_{11} + H^+$	728.7689	0.0010
			742.7831	$C_{41}H_{95}N_{11} + H^+$	742.7845	0.0014
			756.7982	$C_{42}H_{97}N_{11} + H^+$	756.8002	0.0020

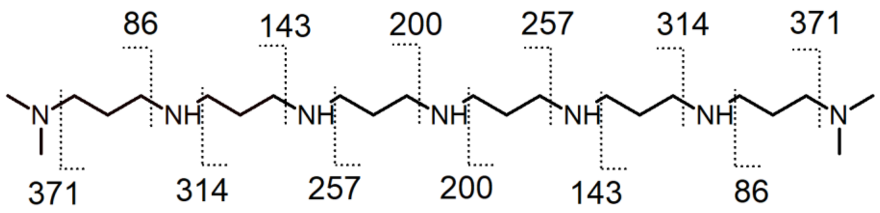

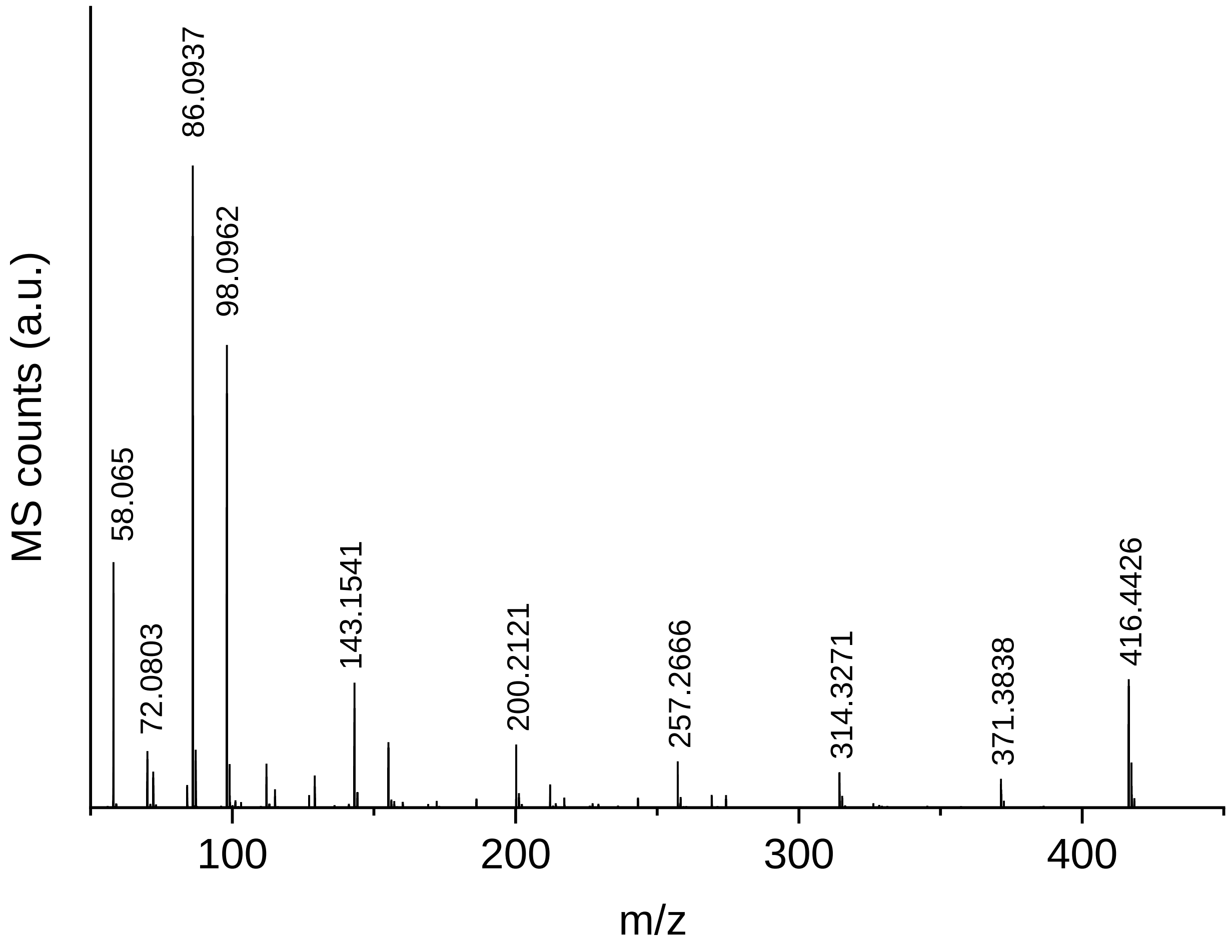

Fig. S3 Molecular structure and fragmentation spectrum of m/z 416.4426 (LCPA fraction 3)

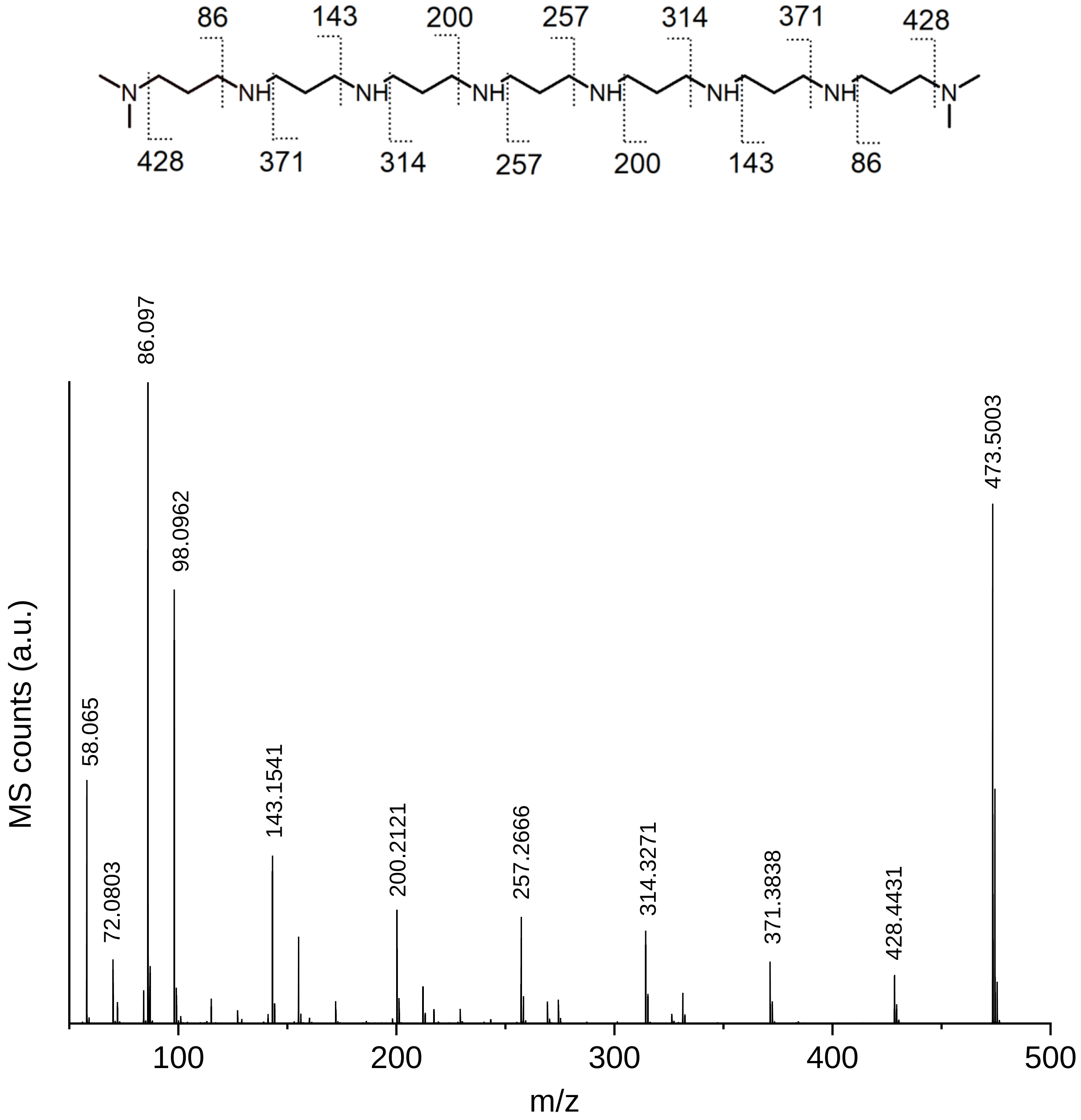

Fig. S4 Molecular structure and fragmentation spectrum of m/z 473.5003 (LCPA fraction 4)

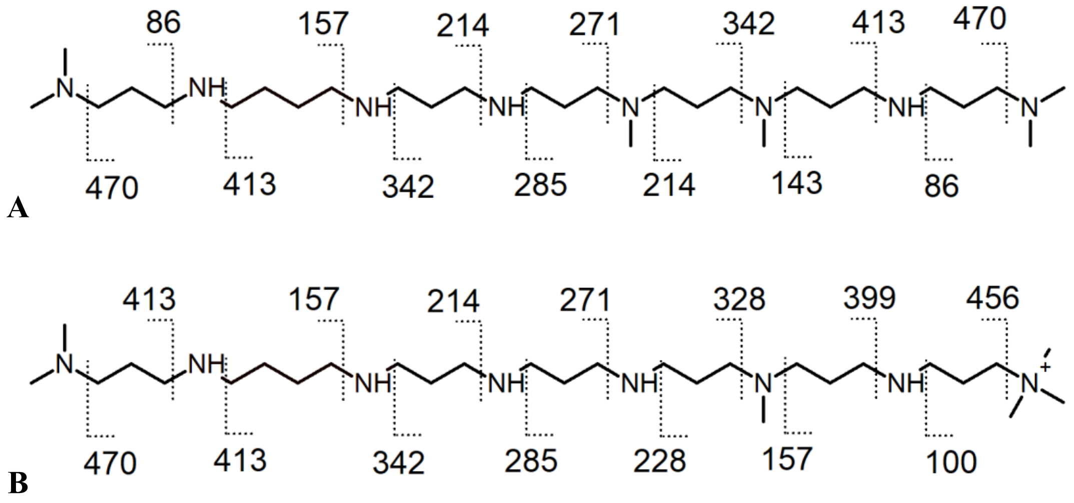

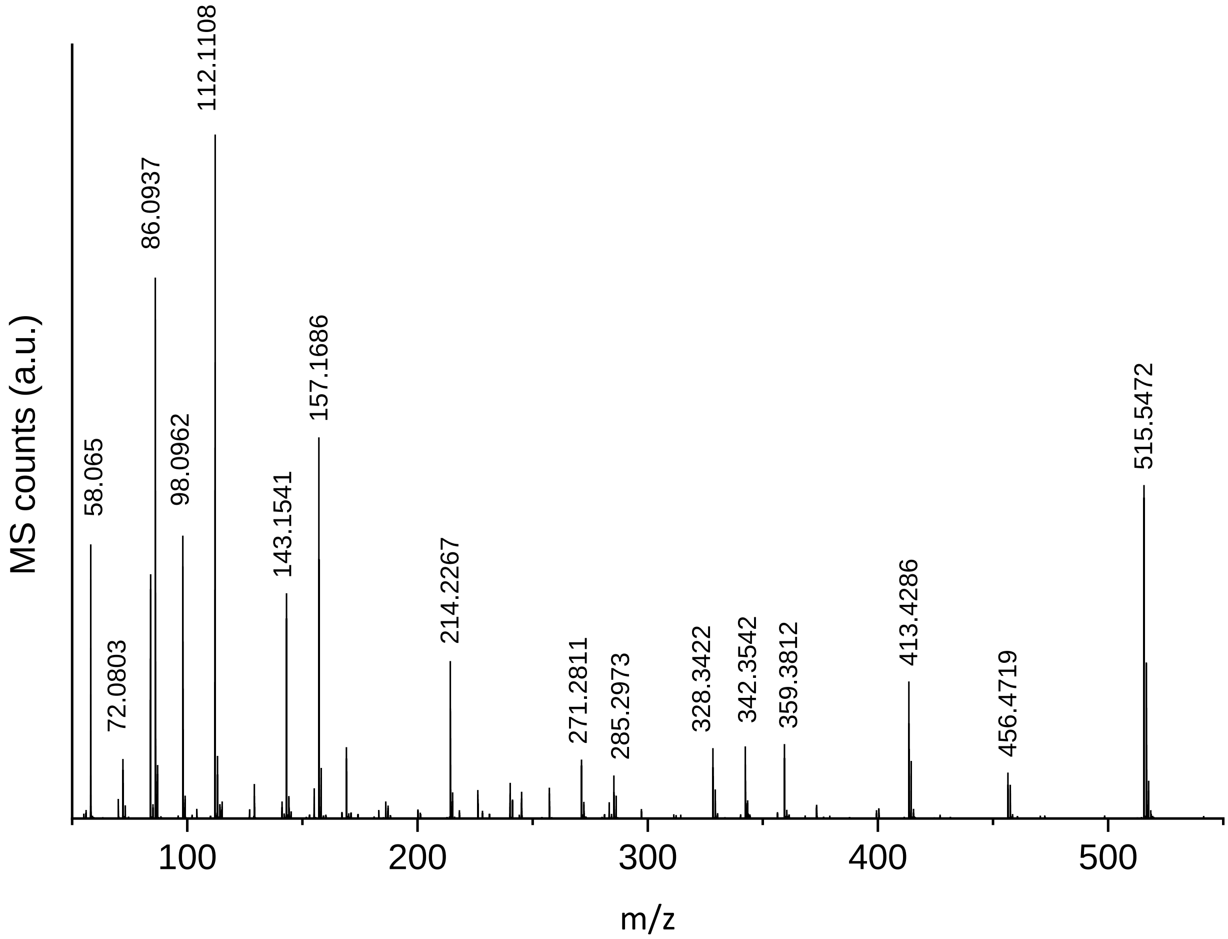

Fig. S5 Molecular structure A and B and fragmentation spectrum of isomeric m/z 515.5472 (LCPA fraction 4)

Analysis of biosilica of *Synura echinulata*

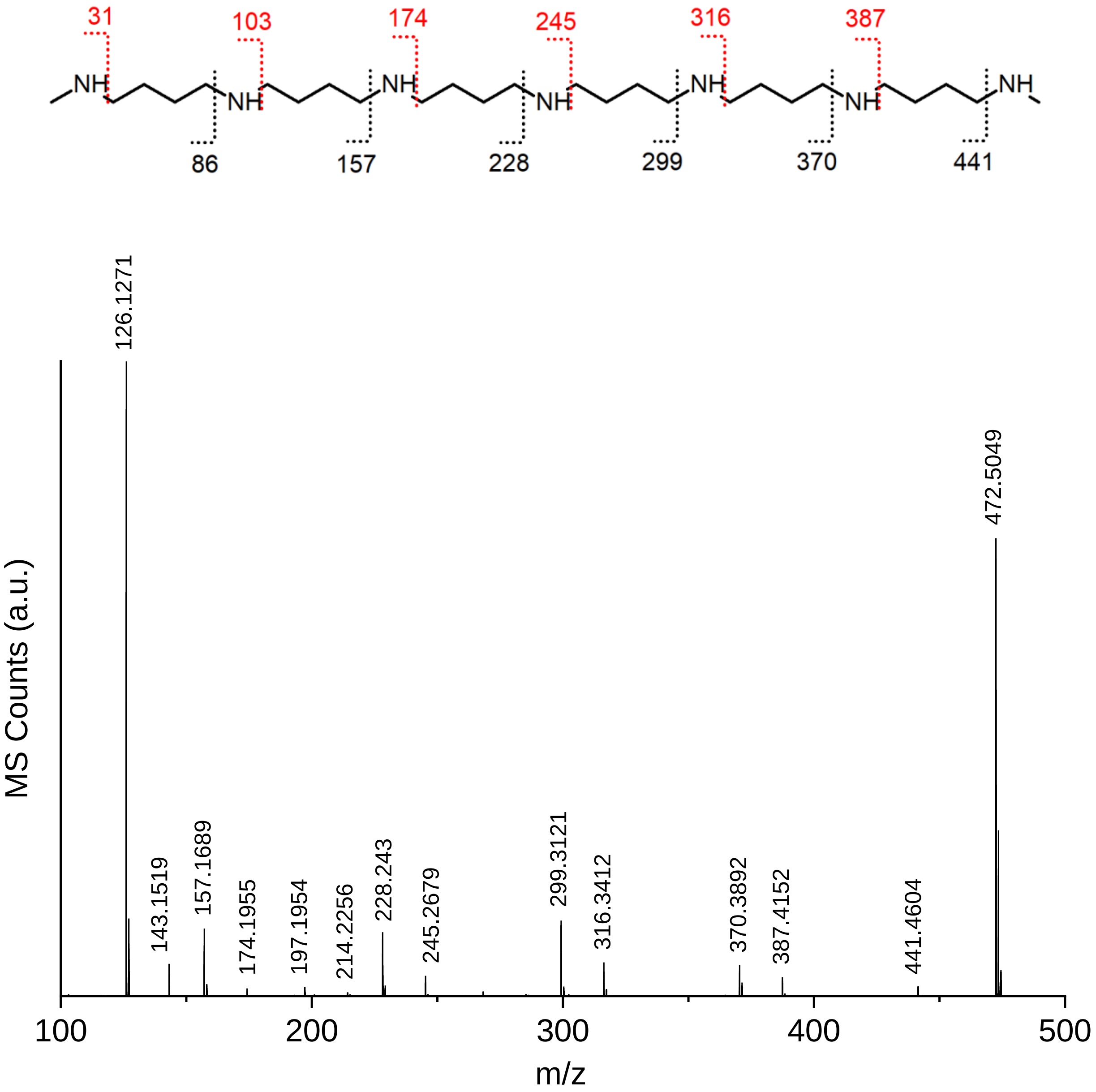

Fig. S6 Molecular structure and fragmentation spectrum of m/z 472.5049 (LCPA fraction 1)

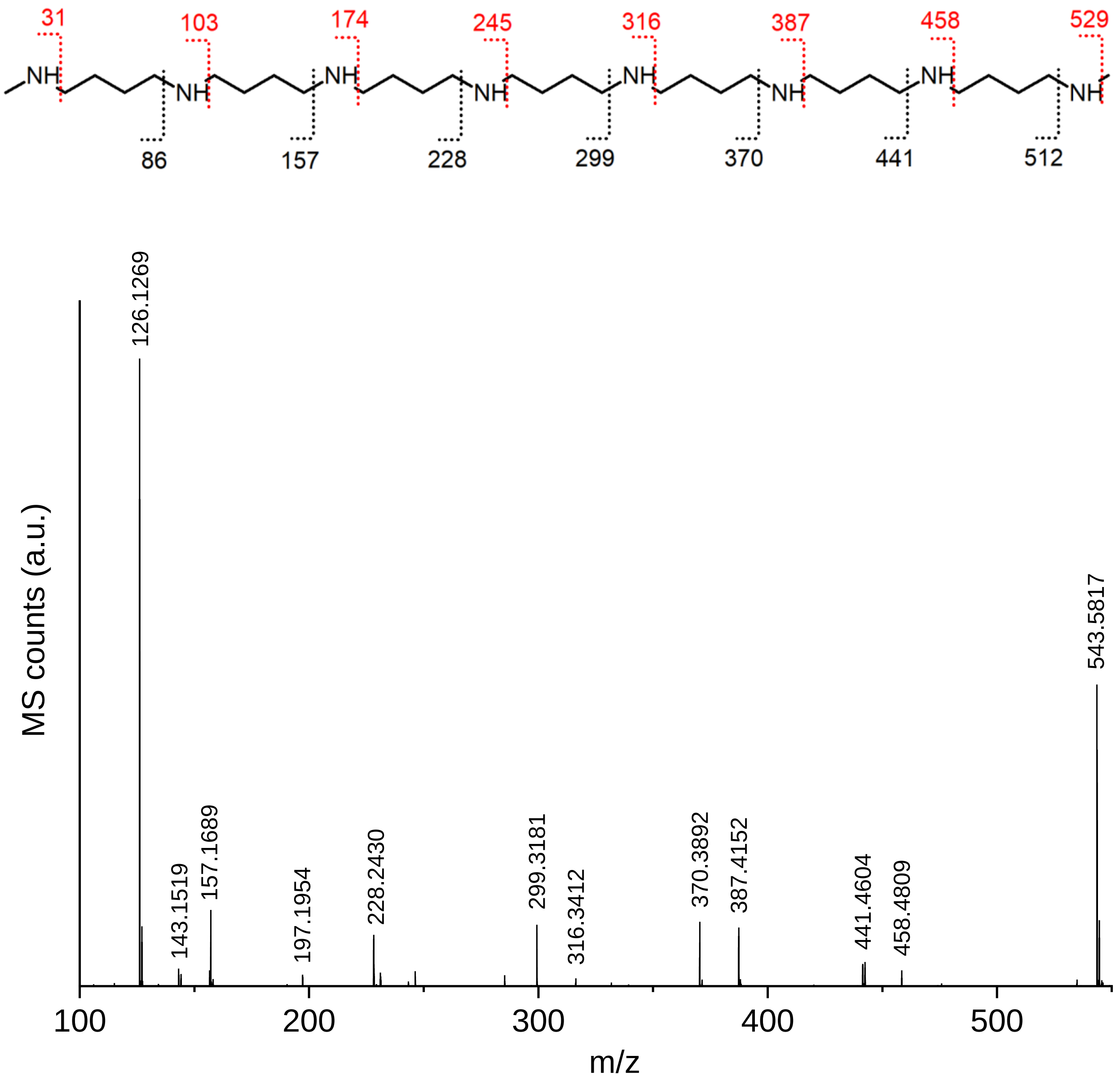

Fig. S7 Molecular structure and fragmentation spectrum of m/z 543.5817 (LCPA fraction 2)

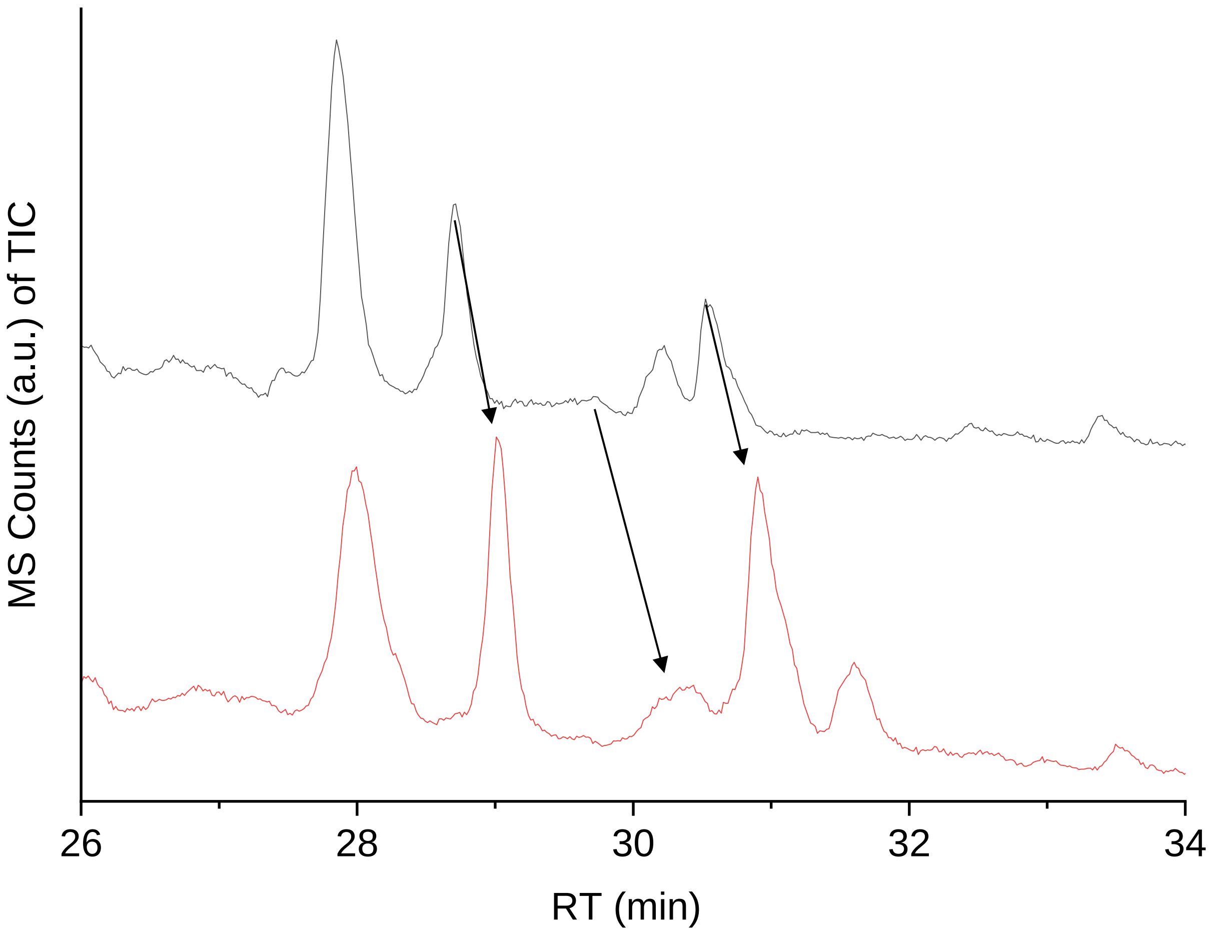

Fig. S8 Results of the reductive methylation of the LCPAs: HPLC-MS (TIC) chromatogram of the re-dissolved remnant after HF treatment of lysed *S. echinulata* cell walls before (black) and after (red) reductive methylation. A shift to higher retention times is well observable due to the more unpolar character of the permethylated LCPAs

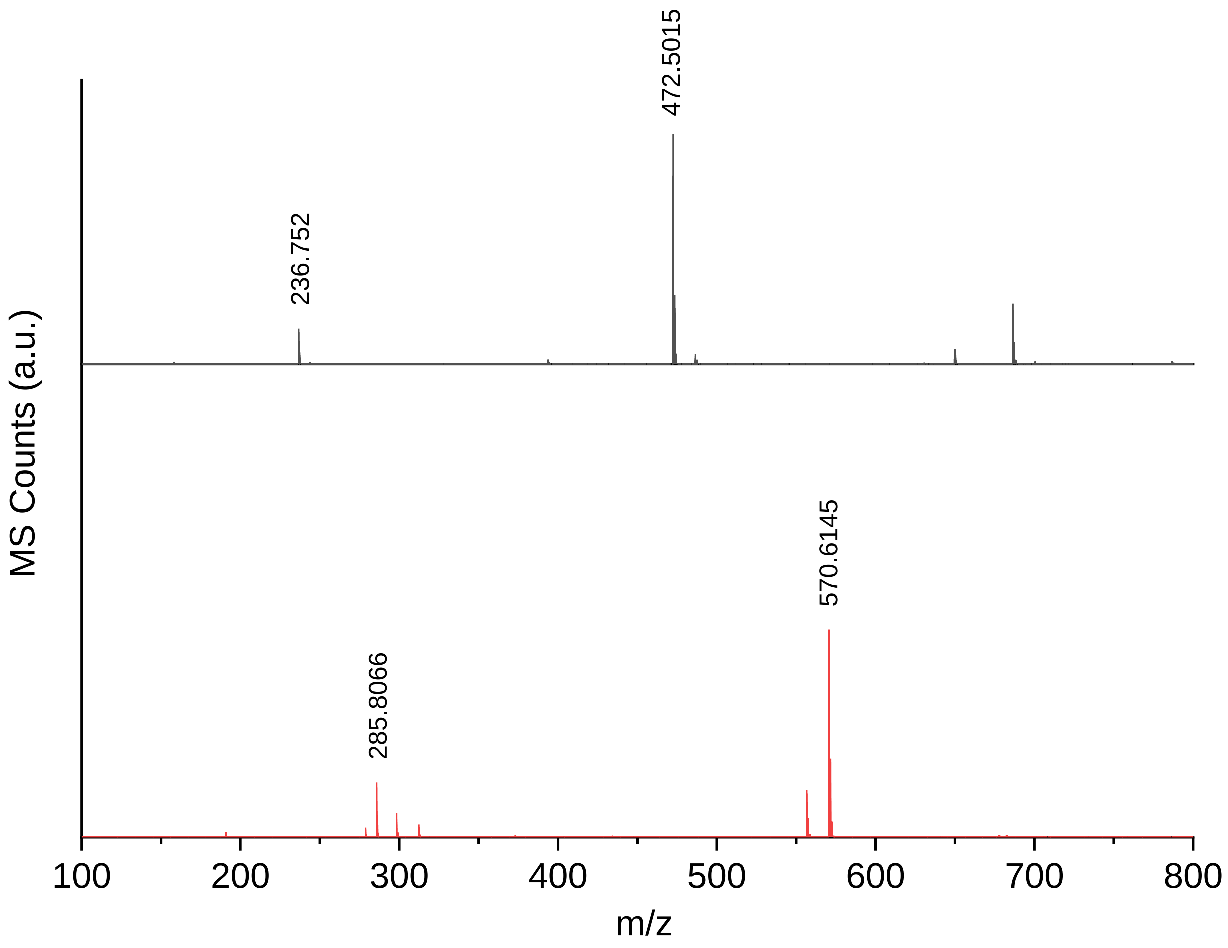

472.5015
236.752
570.6145
285.8066
MS Counts (a.u.)
100
200
300
400
500
600
700
800
m/z

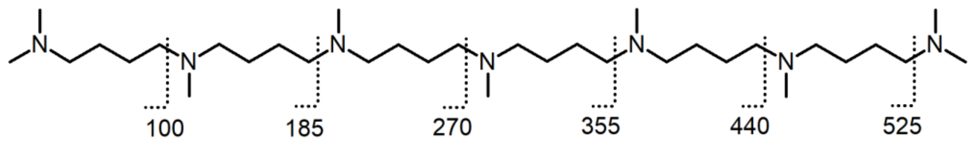

N
100
185
270
355
440
525

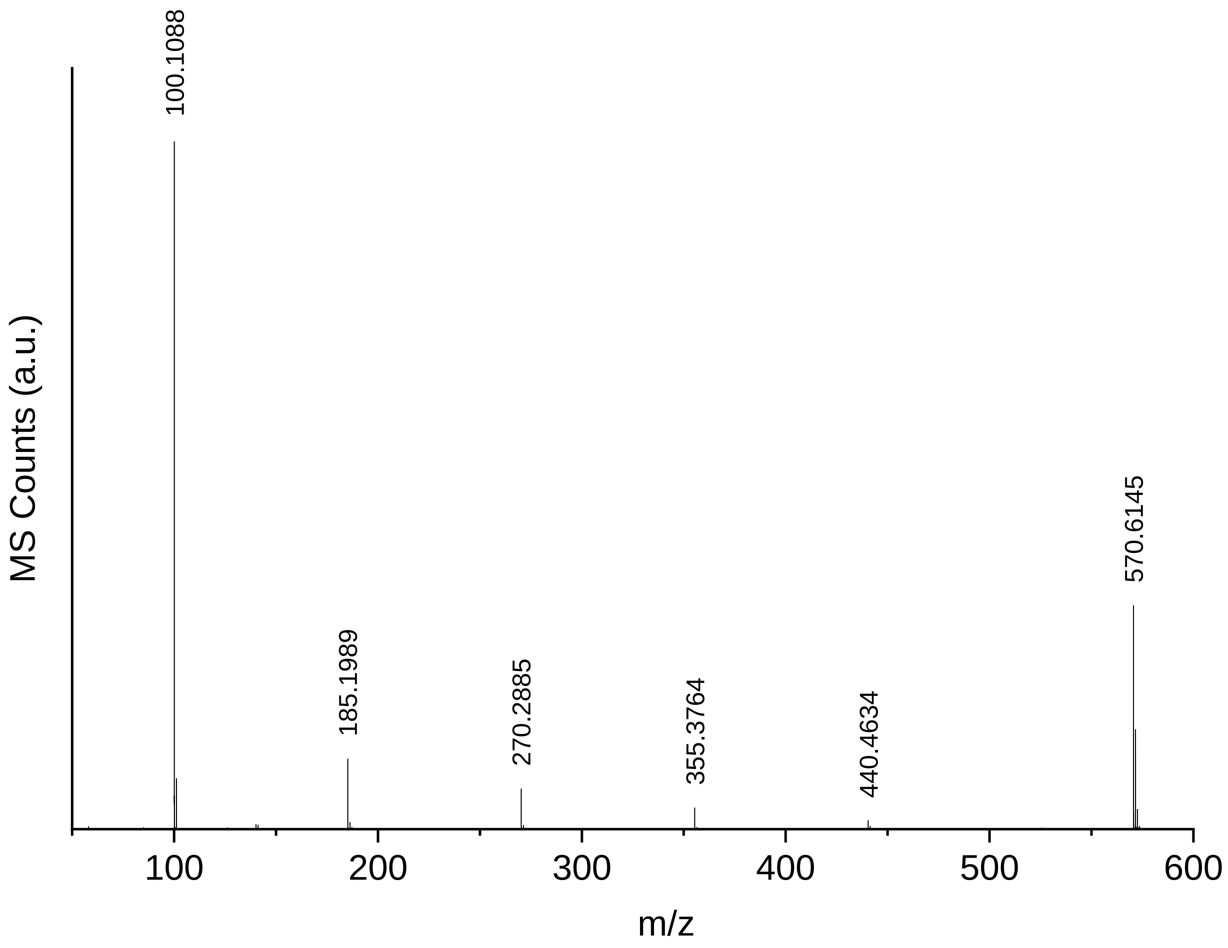

Fig. S9 Results of the reductive methylation of the LCPAs. Top (p.16): MS Spectrum of LCPA fraction 1 before (black) and after (red) reductive methylation. A shift from m/z 472.5015 to m/z 570.6145 is observable. Bottom (p.16): Molecular structure of the methylated LCPA with m/z 570.6145. Top (p.17): MS/MS fragmentation spectrum of m/z 570.6148. A simple fragmentation pattern with a constant interval of m/z 85.089 due to the loss of N-methyl amino butyl is observable

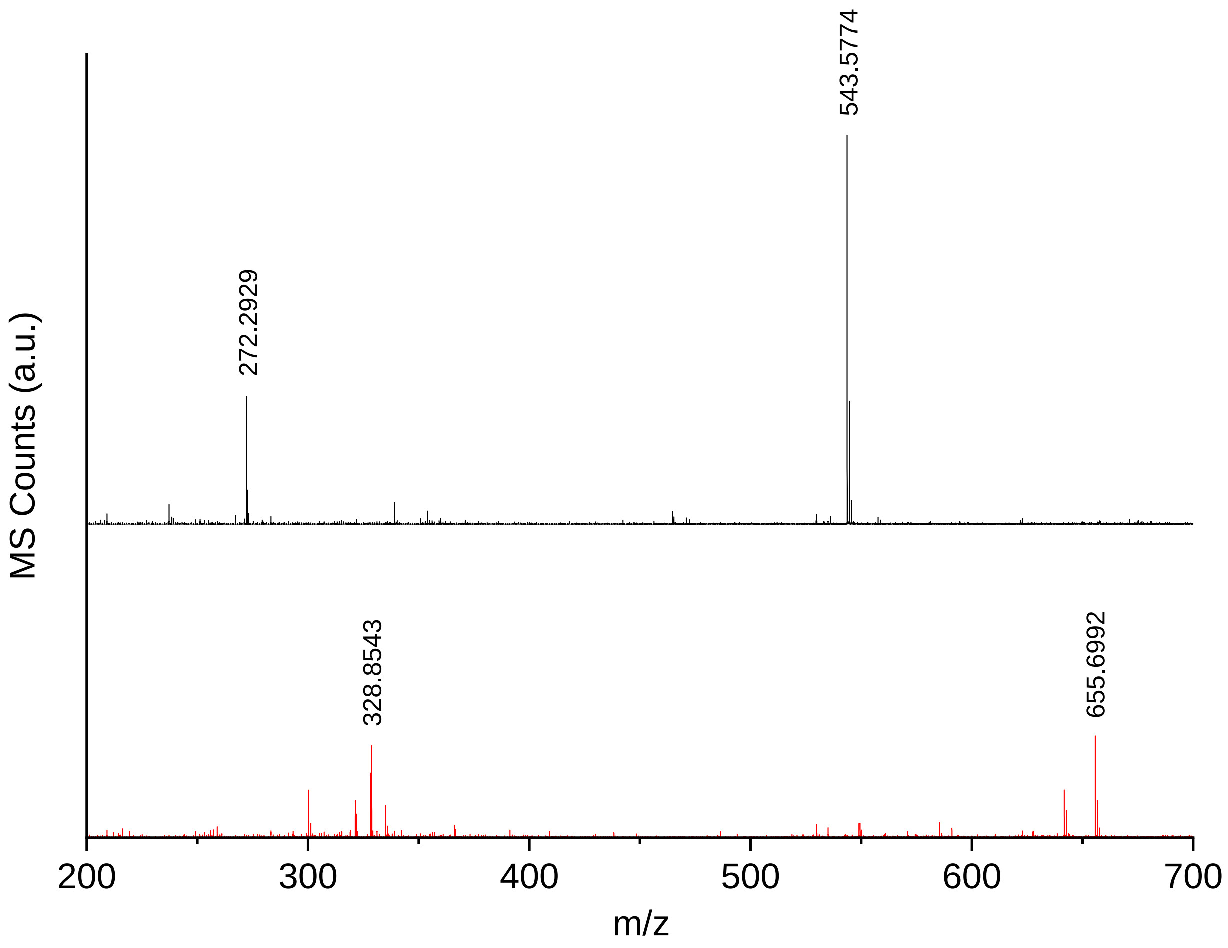

543.5774
272.2929
328.8543
655.6992
MS Counts (a.u.)
200
300
400
500
600
700
m/z

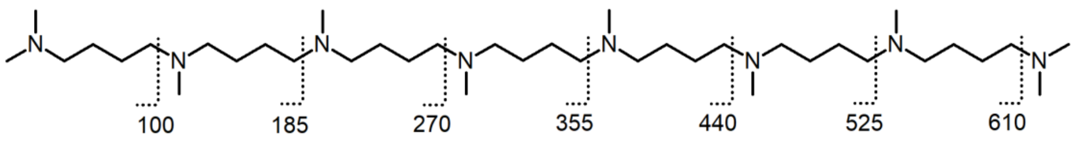

100
185
270
355
440
525
610

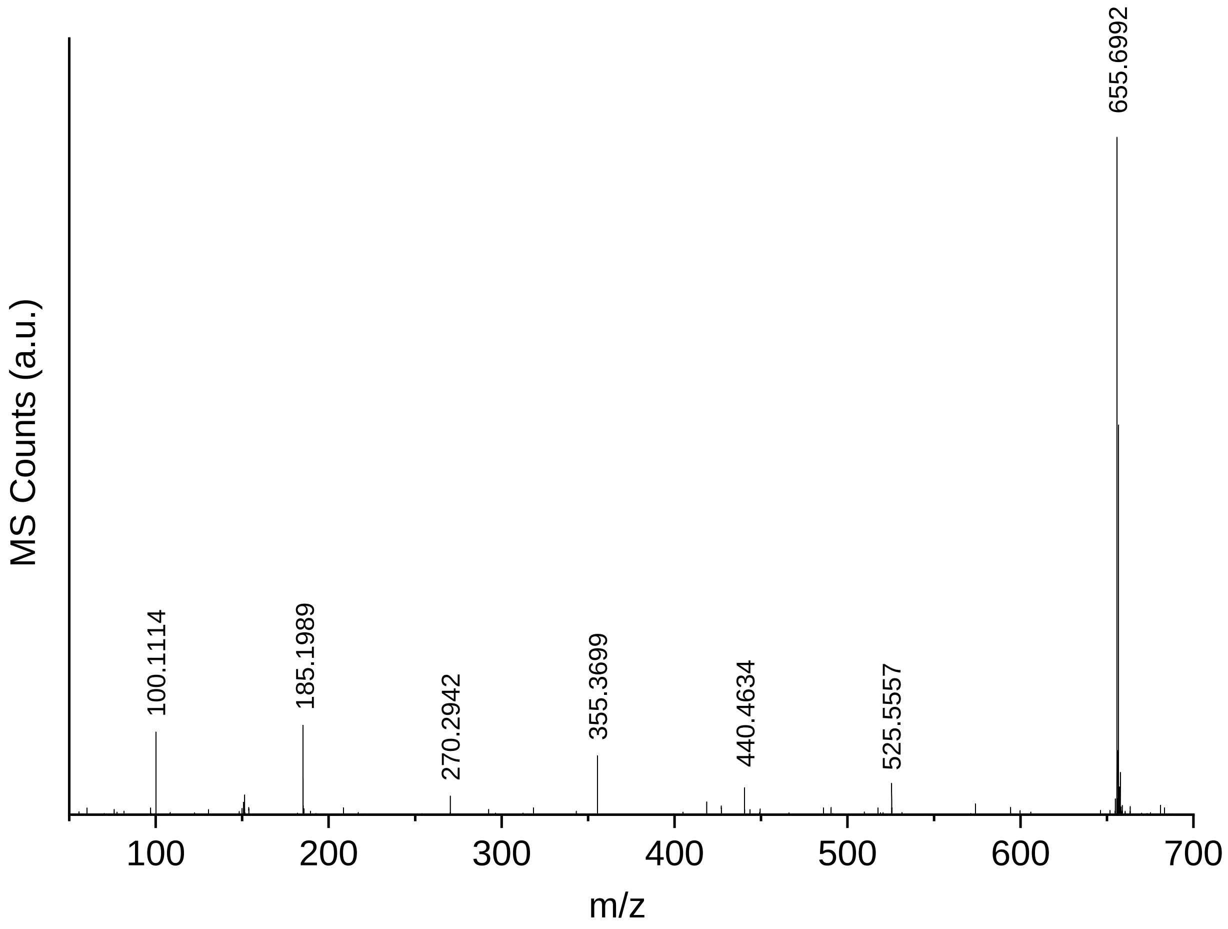

Fig. S10 Results of the reductive methylation of the LCPAs. Top (p.18): MS Spectrum of LCPA fraction 2 before (black) and after (red) reductive methylation. A shift from m/z 543.5774 to m/z 655.6992 is observable. Bottom (p.18): Molecular structure of the methylated LCPA with m/z 655.6992. Top (p.19): MS/MS fragmentation spectrum of m/z 655.6996. A simple fragmentation pattern with a constant interval of m/z 85.089 due to the loss of N-methyl amino butyl is observable

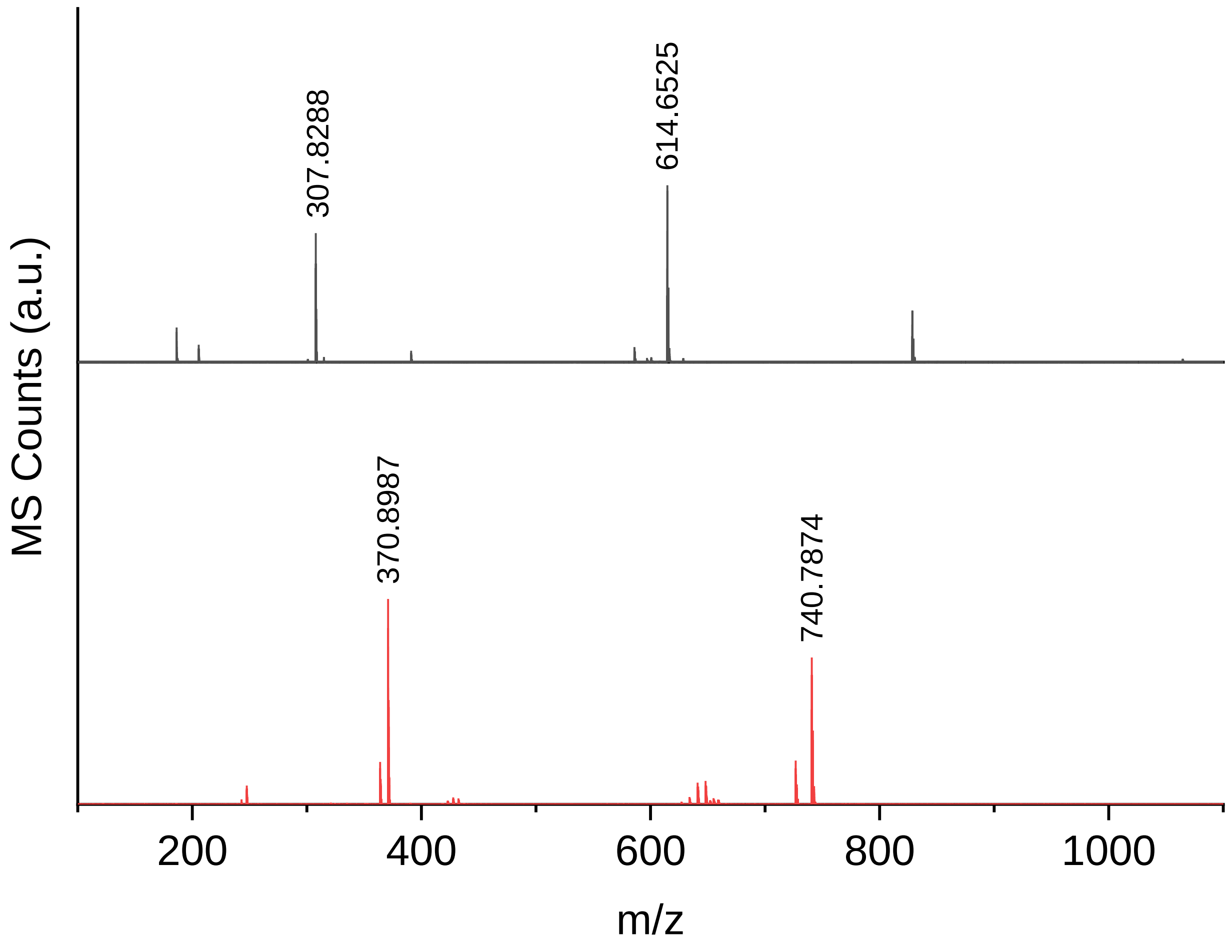
307.8288
614.6525
370.8987
740.7874
MS Counts (a.u.)
200
400
600
800
1000
m/z

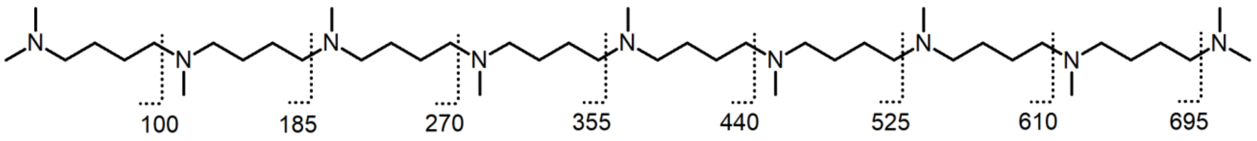
N
100
185
270
355
440
525
610
695

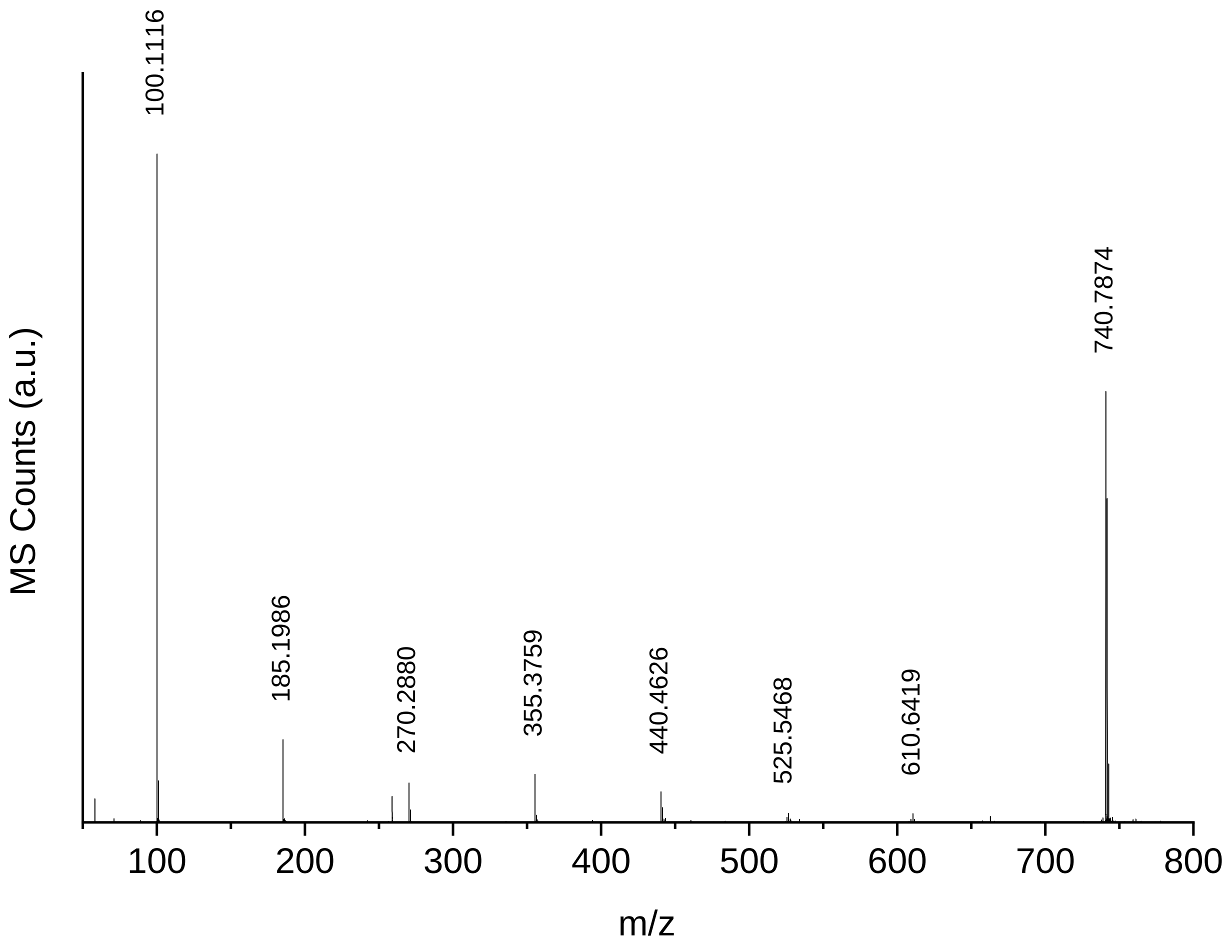

Fig. S11 Results of the reductive methylation of the LCPAs. Top (p.20): MS Spectrum of LCPA fraction 3 before (black) and after (red) reductive methylation. A shift from m/z 614.6525 to m/z 740.7874 is observable. Bottom (p.20): Molecular structure of the permethylated LCPA with m/z 740.7874. Top (p.21): MS/MS fragmentation spectrum and fragmentation pattern of m/z 740.7874 (LCPA fraction 3 after reductive methylation). It shows a simple fragmentation pattern with a constant interval of m/z 85.089 due to the loss of N-methyl amino butyl
